# Developing the New Kent Complex Psychosis Service (KCPS): Reducing the Limitations Imposed by Treatment-Resistant Psychosis

**DOI:** 10.1192/bjo.2024.456

**Published:** 2024-08-01

**Authors:** Aderopo Adelola, Eromona Whiskey, Patricia Morgan, Agostina Secchi, Hayley Stentzel

**Affiliations:** 1 Kent and Medway NHS and Social Care Partnership Trust, Canterbury, United Kingdom.; 2 Kent and Medway Medical school, Canterbury, United Kingdom

## Abstract

**Aims:**

A third to a half of patients with psychosis fail to recover to premorbid levels of functioning. Within these are a group of patients with treatment-resistant psychotic disorders, whose presentations are complex, with significant comorbidities, prolonged hospital admissions, and poor social and occupational functioning. Reports suggest an underutilization of clozapine, which is the licensed treatment for resistant schizophrenia for reasons ranging from prescribers’ expertise or reluctance to intolerable side effects and comorbid psychiatric or medical conditions. In Kent, Surrey, and Sussex, clozapine prescription is only 4.93%, which is the third lowest among NHS England Regions.

Complex psychosis in Kent and Medway NHS Partnership and Social Care Trust (KMPT) was handled through a referral through the Out-of-Area Treatment panels to the South London and Maudsley (SLAM) Psychosis unit. This had lengthy wait time for admission and required approval for out-of-area costs which can be significant for longer admissions, placed a considerable travel burden on the family/carers, and made it difficult for reintegration into the local community.

**Methods:**

The KCPS was set up as a consultation service to ensure that patients receive the right care to facilitate recovery and that our healthcare professionals and teams are supported in meeting the needs of patients with complex psychosis. This multidisciplinary service, comprising psychiatrists, pharmacists, occupational therapists, and administrators, commenced functioning in January 2023 and we examined the first year of operation. KCPS reviewed the detailed psychiatric/medication history, highlighting prior treatment and effectiveness, with a focus on doses, tolerability, duration, and adherence; we explored the social, occupational, and psychological functioning of each patient; liaised with referrers/carers, reviewed the relevant research literature and provided holistic recommendations to the referrers.

**Results:**

From January to December 2023, there were 36 referrals from a mixture of services, 26.3% of these were from acute wards. The patient's mean age was 42.8 years; 75% were male; the most common diagnosis was schizophrenia (50%), and the commonest comorbidities were Autism spectrum disorder and diabetes (13.9% and 27.8% respectively). Feedback from referrers and carers reported a high level of satisfaction with the service.

**Conclusion:**

Reasons for referral included diagnostic uncertainty, comorbidity, intolerable side effects of clozapine leading to its early discontinuation, and poor psychosocial functioning. The KCPS recommendations were deemed useful in changing the trajectory of illness in some individuals, leading to early discharge and avoiding an out-of-area placement for treatment. Professionals appreciated the opportunity to discuss complex cases in a supportive, friendly, and in-house environment.

